# The impact of government subsidy programs on equity in health financing

**DOI:** 10.1186/s12962-023-00460-w

**Published:** 2023-08-14

**Authors:** Yousef Mohammadzadeh, Aysan Sheikhmali, Jafar Yahyavi Dizaj, Ali Mohammad Mosadeghrad, Hasan Yusefzadeh, Arash Refah Kahriz

**Affiliations:** 1 Department of Economics, Faculty of Economics and Management, Urmia University, Urmia, Iran; 2 Department of Economics, Faculty of Economics and Management, Urmia University, Urmia, Iran; 3 Department of Health Management & Economics, School of Public Health, Tehran University of Medical Sciences, Tehran, Iran; 4 School of Public Health, Tehran University of Medical Sciences, Tehran, Iran; 5 Department of Management and health economics, Faculty of Health, University of Medical Sciences, Urmia, Iran

**Keywords:** Health system evolution plan, Justice in the healthcare system, Fair financial contribution index, Targeted subsidy plan

## Abstract

**Background:**

Iran government implemented the targeted subsidy plan in December 2010 to reduce energy consumption and inequality. In addition, the Health Transformation Plan was implemented by the Ministry of Health to reduce out-of-pocket payments. This study aimed to examine the impact of these two government subsidy programs on equity in health financing.

**Method:**

In this study, data on 528,046 households were collected using household surveys during 14 years (2007–2020). The Fairness in Financial Contribution index and Catastrophic Health Expenditures index were calculated. Also, a Logistic regression model was performed by the applied software of Stata V.14 to examine the effects of the two mentioned policies and other socioeconomic characteristics of households on their exposure to Catastrophic Health Expenditures.

**Results:**

The FFC index was 0.829 and 0.795 respectively in 2007 and 2020. The trend analysis did not show significant changes in the FFC index between 2007 and 2020. TSP and HTP implementations do not reduce households’ exposure to CHE significantly. Crowded households with more elder people, belonging to low-income deciles, without houses, and living in rural areas and deprived provinces, are more likely to be at risk of CHE. Health insurance coverage did not protect households from CHE. Highly educated and employed households were exposed to less CHE.

**Conclusion:**

The government subsidy programs have not been effective in improving FFC and reducing CHE indices. None of them has been able to realize the goal of the 6th National Development Plan of reducing CHE to 1%. The government should devise support packages for target households (households with more elderly people, lower incomes, without private houses, crowded, rural, and inhabited in deprived provinces), so they can protect households against CHE. Modifying and improving the quality of insurance coverage is strongly recommended due to its inefficiency.

## Background

The health system has an important role in changing the health status of individuals; this role is played in the form of the provision of preventive, therapeutic, and sanitary services [38]. The acceptance of health as all individuals' right, which should be achieved at the highest level, makes governments obligated and committed to the treatment and prevention of illnesses so that they make all their efforts to create a situation where access to health services is available to all people [12, 13]. Measures like granting subsidies, reducing inequality, and observing justice in delivering health services [19], as well as fair financing, can play an important role in improving the performance of the health system in providing the best quality services [3]. According to the World Health Organization (WHO, the statement "you get what you pay for" refers to the same concept of justice in the market transactions while the health system is different from the other conventional markets. People should have access to health services regardless of their financial and economic status to continue their lives and maintain their mental and physical health standards, and lack of purchasing power should not prevent them from receiving services. This is because, firstly, health care is expensive inherently and secondly, the need for health services is unpredictable. Therefore, the health system financing should be fair in a manner that people do not face catastrophic expenditures when they need health services. Individuals should consider a percentage of their household income as payment for receiving these health services, these payments may impose catastrophic costs on some households and bring them below the poverty line [1, 19, 32].

According to WHO, households face catastrophic health expenditures when their healthcare cost equals or exceeds 40% of the total household capacity. As a result, these households may discontinue receiving health promotion services and prefer to tolerate illnesses, or they may disregard their basic needs such as education, clothing, etc. [38]. Catastrophic payments are very common in developing countries with moderate levels of income and low-income countries [39]. Given the vital role of financing in the health system, fair financial contribution has become one of the most important goals and concerns of the health systems (Murry, 2000). In addition, people's access to health care, inequality in responsiveness, and inequality in health care are largely influenced by health system financing.

The Islamic Republic of Iran is a middle-income country with a population of about 78 million, annual population growth of 1.28%, and a median age of 29 years. The gross national income per capita is (PPP int.) $17,400. Seventy-two percent of the population lives in urban areas [10].

The Iranian healthcare system consists of public, private, and non-government organizations (NGO)-funded healthcare. The Ministry of Health and Medical Education (MOHME) is responsible for policy-making, financing, planning, and controlling the health sector at the national level. At the provincial level, medical universities are responsible for providing both medical education and healthcare services. The district health network provides primary healthcare (PHC) services free of charge, and the hospital network delivers secondary and tertiary services [18].

Government general revenues (e.g., taxes), public and private health insurance premiums, and individuals’ out-of-pocket (OOP) payments are the main sources of financing health systems. The health financing system in Iran is somewhat backward, fragmented, inefficient, and inequitable. Formal workers and their dependents are insured by the Social Security Organization (SSO), and members of the military and their dependents are covered through the Armed Forces Medical Service Organization (AFMSO). The remainder of the population is eligible to enroll in the Iran Health Insurance Organization (IHIO), which covers government public sector employees, rural households, the self-employed, clerics, students, and so on [18].

Fair health system financing influences access and equity in the health system. Fair financial contribution is an important goal of the health system. Households' contributions in financing health expenditures determine the fairness of health system financing. Fair financial contribution (FFC) and catastrophic health expenditures (CHE) are examples of indicators used for calculating equity in financing the health system. Iran devoted 6.6% of its gross domestic product to total health spending (1218 PPP int. $ per person) in 2012. The private expenditure on health as % of total expenditure on health was 59.6% of which 88% was out of pocket [36].

Iran has made good progress in improving population health outcomes during the last three decades. Communicable diseases are well controlled; however, the country faces a burden of non-communicable diseases in addition to an increase in physical accidents and injuries due to the growth of urbanization and industrialization. The Iranian health system still faces several challenges when it comes to access, equity, quality, and efficiency. As a result, some healthcare reforms and initiatives have been implemented to enhance the referral system, increase capacity for training healthcare personnel, expand access to healthcare services, reduce inequities, and promote the quality of healthcare services.

Governments implement subsidy reform, besides environmental concerns, to increase national welfare by reducing the gap between domestic and reference energy prices. Whether energy demand is elastic and the current price approaches the reference price; reducing energy consumption on the internal market, thus reducing both national energy costs and local and global environmental damage. In developing countries, where there is no reliable and up-to-date information infrastructure on household income, energy subsidies are particularly frequent. Discrimination in the area of compensatory reimbursement can induce people to under-report their income. An appropriate alternative could be direct deposit to all consumers. The reform plan was implemented in Iran in December 2010, and the government has decided to make a major adjustment to energy subsidies. Remove the unequal distribution of subsidies and decrease energy consumption by changing the distribution of subsidies, energy reforms were implemented by the government to promote more equal distribution of resources, but they were not meant to support the poor. Unconditional universal cash transfers have relieved the government of the difficulty of identifying vulnerable groups. Two years after this reform plan was implemented, poverty was reduced in absolute and relative terms and income inequality improved slightly. However, the cash transfer could not fully offset the negative impact of eliminating the subsidy. Following a smooth start, the plan encountered several problems, which resulted in a temporary delay in the second phase of the reforms. A budget deficit that was far bigger than the national budget was burdensome for the government. These shocks caused inflation and devaluation of the national value were not only linked to the reforms, but also to the changes in the economy. International sanctions have played a significant role in the economic instability that has followed the reforms [2, 40].

The parliament approved the Targeted Subsidies Plan (TSP) in 2010 and asked the government to replace subsidies on energy and food with targeted social assistance. The Government implemented the targeted subsidy plan to reduce energy consumption. Improving the distribution of energy subsidies by replacing them with universal cash transfer was the by far the second goal. The removal of subsidies resulted in an increase of about 21% in prices. The amount of the universal cash transfer was 455,000 Rials (approximately $ 41 in 2010 and $ 1.5 in 2022) and remained the same over these 11 years. The government was also asked to use the freed funds for expanding social insurance, providing healthcare services, promoting community health, and covering severely ill patients' treatment and medicines. TSP was part of a broader Iranian economic reform plan based on the country’s 5-year economic development plan. The government implemented the plan in 2010 [5].

Spending on TSP exceeded the additional revenue from the reform of energy prices (which was previously subsidized). Because energy consumption was lower without the subsidies, and international oil prices fell. Of course, the international oil price of oil also fell [29]. In the first eighteen months of this reform, spending on TSP was almost twice the amount of the increase in government revenue that resulted from eliminating the energy subsidies [5]. Thus, in 2014, the government decided to stop paying the top 20% of rich households direct cash due to the budget limit.

Later on, the Ministry of Health and medical education implemented a series of reforms, called the Health Transformation Plan (HTP) to expand access to healthcare services, promote equity, reduce the catastrophic and impoverishing OOP payments, and improve the quality of healthcare services. The HTP was mainly focused on three departments of the MOHME (i.e., curative care, health, and education). Accordingly, all uninsured people were encouraged to register in the IHIO. All of the MOHME-affiliated hospitals (561 out of the total 878 hospitals) should provide all necessary inpatient services. Patients' OOP payments at these hospitals should be less than 10% of the total medical expenditure. The national tariff for medical services was increased in October 2014 to encourage medical consultants to work full-time in public hospitals and provide high-quality services, persuade medical doctors to stay in deprived areas, and reduce informal and illegal payments. The major source of the HTP funding was a raise in the MOHME budget comprising 1% of the value-added tax (VAT) and 10% of freed subsidies [24].

It is necessary to measure the effectiveness of these two government subsidy programs. Hence, this study aimed to examine the effect of TSP and HTP subsidy programs on the equity of financing healthcare services in Iran.

## Method

The data of this retrospective and descriptive study was obtained from raw data of the annual survey of household income and expenditure conducted by the Iran Statistical Center (ISC, 2007–2020). The statistical population of the study consisted of all Iranian households.^1^ The randomized three-stage cluster sampling method was used for selecting samples [22]. The "Household Income and Expenditure Survey" questionnaires were completed by interviewing the head of households. The questionnaire covered questions about the social characteristics of the household members, household properties, food and non-food expenditures, and household income. Access 2019, Excel 2019, and STATA, v.17 were used to organize and analyze data. 528,046 households from 2007 to 2020 (including 265,310 rural and 262,736 urban households) participated in this study. This data is collected from different households every year, so this study used the total data collected over 14 years. Therefore, these data are cross-sectional. Table 1 shows the sample size for each year.

**Table 1:** The survey sample size (2007–2020)

Year	Rural	Urban	Total	Year	Rural	Urban	Total
2007	16,266	15,019	31,285	2014	19,391	18,886	38,277
2008	19,708	19,382	39,090	2015	19,382	18,872	38,254
2009	18,204	18,666	36,870	2016	19,340	18,809	38,149
2010	19,585	18,702	38,287	2017	19,261	18,701	37,962
2011	19,787	18,728	38,515	2018	18,610	20,350	38,960
2012	19,658	18,536	38,194	2019	18,430	19,898	38,328
2013	19,437	18,881	38,318	2020	18,251	19,306	37,557

Source: Isc, 2007–2020

In this study, Fair Financial Contribution (FFC) index and Catastrophic Health Expenditures (CHE) index were calculated for measuring equity in healthcare financing. The FFC index is an indicator of financial equity which varies between 0 and 1; the fairer the health financing system, the closer the FFC index to 1. Mode 1 is the ideal state of the FFC index in the health system [35]. The World Health Organization formula was used for calculating the FFC index in the health system [19].

$$FFC=1- \sqrt[3]{\frac{\sum_{h=1}^{n}{w}_{h}/{oopctp}_{h}-{oopctp}_{0}/3}{\sum {w}_{h}}}$$

where W_h_ is the household weighting variable when sampling with the actual population ratio is different in the rural and urban areas (h: the household identification code); OOPCTP_h_ (OOP_h_/CTP_h_) is the household's out-of-pocket payments for the health care services divided by the capacity to pay; OOPCTP_0_ is calculated by dividing the total household health expenditure by the total capacity to pay. The CHE index was set at 40% or higher of the household capacity to pay [38]. That is, if households spend more than 40% of their capacity on healthcare services, they suffer from CHE [20].

Based on the theoretical literature and empirical studies on the determinants of catastrophic health expenditures (such as [7, 21, 9]), as well as the identification of new variables and indicators in this study (which in the Cost-Income Household survey was available), finally, the following model has been considered to investigate the impact of targeted subsidies on the catastrophic health expenditures of Iranian households.

$$Cata=\alpha +\beta 1TSP+\beta 2Insurance+\beta 3HTP+\beta 4Size+\beta 5Develop+\beta 6R\_U+\beta 7lnum+\beta 8Empnum +\beta 9 Decile +\beta 10Housing+\beta 11Elder$$

- Cata: Catastrophic Health Expenditure*s* calculated as, CHE = 1 if $OOP/CTP\hspace{0.17em}\ge \hspace{0.17em}0.4$, otherwise CHE = 0 that takes values of zero and one; therefore, the dependent variable is a binary variable and we will use logistic models to estimate it; Insurance: Households with or without health insurance coverage.
- HTP: Health Transformation Plan (Zero before the project (until 2013) and number one for the period after the project (2014–2020)).
- Size: Size of households (population of households).
- Develop: Development status of the province where the household resides.^2^
- R_U: Household residence (town or village).
- Lnum: Number of literate people in the household.
- Empnum: Number of people working in the household.
- Decile: the household expenditure decile.^3^
- Housing: Homeownership status.
- Elder: Number of elderly people in the household.^4^

## Results

The FFC index in 2007 and 2020 was 0.829 and 0.795, respectively. The average FFC index for the years 2007–2013 was 0.827 and for the years 2014–2020 was 0.82. Therefore, trend analysis does not show significant changes in the FFC index. Of course, the trend of the index from 2013 to 2017 has been almost constant, but in the last three years, it has decreased slightly. TSP increased FFC by 9.6% (from 0.781 in 2010 to 0.856 in 2011) and HTP increased it by 0.5%. But in later years they could not improve. In recent years, Iran's economy has faced economic sanctions, devaluation, and high inflation, and as a result, the FFC index has declined (Table 2).

**Table 2:** Descriptive of the FFC index in the healthcare sector between 2007 and 2020

Year	Rural	Urban	Total	Year	Rural	Urban	Total
2007	0.817	0.841	0.829	2014	0.821	0.841	0.831
2008	0.82	0.842	0.831	2015	0.821	0.842	0.831
2009	0.802	0.835	0.818	2016	0.82	0.84	0.83
2010	0.785	0.778	0.781	2017	0.82	0.84	0.83
2011	0.846	0.866	0.856	2018	0.81	0.83	0.82
2012	0.838	0.863	0.85	2019	0.80	0.80	0.80
2013	0.814	0.841	0.827	2020	0.80	0.79	0.795

As it is shown in Table 3, about 2.5% of households suffered from CHE in 2007, the figure increased to 3.3% in 2010, then dropped to 1.8% in 2011. Then, it experienced a rising trend and reached 4.11% in 2020. The mean of the CHE index was 2.9% for the years 2007–2010 and 3.2% for 2011–2017 and 3.85% for the years 2018–2020.

**Table 3:** Descriptive of households suffering CHE between 2007 and 2020

Area of residence	Rural	Urban	Total
	Number (Percentage)	Number (Percentage)	Number (Percentage)
2007	494 (3%)	305 (2%)	798 (2.5%)
2008	663 (3.3%)	418 (2.1%)	1081 (2.7%)
2009	660 (3.6%)	469 (2.5%)	1129 (3%)
2010	762 (3.8%)	527 (2.8%)	1289 (3.3%)
2011	467 (2.3%)	246 (1.3%)	713 (1.8%)
2012	481 (2.4%)	271 (1.4%)	752 (1.9%)
2013	840 (4.3%)	537 (2.8%)	1377 (3.5%)
2014	859 (4.4%)	510 (2.7%)	1369 (3.5%)
2015	824 (4.2%)	511 (2.7%)	1335 (3.4%)
2016	815 (4.2%)	500 (2.7%)	1315 (3.45%)
2017	839 (4.3%)	555 (3%)	1394 (3.65%)
2018	835 (4.49%)	554 (2.72%)	1389 (3.57%)
2019	886 (4.81%)	601 (3.02%)	1487 (3.88%)
2020	918 (5.03%)	627 (3.25%)	1545 (4.11%)

The number of households facing CHE has increased in recent years. Of course, Iran's economy has faced major crises in recent years. GDP growth per capita in 2018 and 2019 was − 7.3% and − 8% percent. Inflation rose above 40% in 2019 and 2020. The average dollar price increased from 107,830 Rials in 2018 to 171,000 Rials in 2020.

Based on the results of the logit model estimation, TSP and HTP implementation not only reduced households' exposure to CHE, but they also caused numerous economic problems, which increased the likelihood of households' exposure to CHE. The results of the model estimation are reported in Table 4. The results also show that crowded households with more elder people, belonging to low-income deciles, without houses, and living in rural areas and deprived provinces, are more likely to be at risk of CHE. Health insurance coverage did not protect households from CHE. Highly educated and employed households were exposed to less CHE. Households living in less developed provinces were facing more CHE. One of the noticeable results of this study is the inefficiency of health insurance plans in protecting households against CHE. Health insurance companies have not been able to reduce the likelihood of household exposure to CHE.

**Table 4:** Estimation of the logit regression model of factors influencing CHE for the study period (2007–2020)

Variable	Odds ratio	Z statistical	p-value
TSP			
Years of receiving subsidies for Households	Basic variable		
Years of non-receiving subsidies by	1.03 (1–1.05)	3.78	> 0.001
Insurance			
Households without health insurance	Basic variable		
Households with medical insurance	1.14 (1.09–1.18)	4.59	> 0.001
HTP			
Years before the plan	Basic variable		
Years after the implementation of the plan	1.02 (1–1.05)	3.72	> 0.001
Size	0.9 (0.89–0.92)	− 3.74	> 0.001
Develop			
Households living in deprived provinces	Basic variable		
Households living in semi-developed provinces	0.94 (0.90–0.98)	− 2.49	> 0.012
Households in developed provinces	0.9 (0.87–0.95)	− 4.7	> 0.001
R_U			
Rural Households	Basic variable		
Urban Households	0.71 (0.67–0.73)	− 14.2	> 0.001
lnum	0.91 (0.88–0.95)	− 2.19	> 0.03
Empnum			
Households without any employed	Basic variable		
Households with 1 employed person	0.75 (0.71–0.78)	− 11.02	> 0.001
Households with 2 or more employed	0.72 (0.69–0.75)	− 7.06	0.000
Decinc	0.84 (0.8–0.88)	− 12.12	> 0.001
Housing			
Households without a private house	Basic variable		
Households owning a private house	0.89 (0.87–0.92)	− 3.74	> 0.001
Elder			
Households without elderly	Basic variable		
Households of one elder	1.31 (1.28–1.35)	8.05	> 0.001
Households with 2 or more elderly	1.85 (1.8–1.89)	11.34	> 0.001
cons_	0.21 (0.2–0.21)	− 81.41	> 0.001

Log likelihood = − 39,361.972

## Discussion

The Iranian government launched the targeted subsidy plan (TSP) in December 2010 to reduce inequality and poverty. In addition, the Health Transformation Plan (HTP) was implemented by the Ministry of Health to reduce out-of-pocket payments. The study aimed to examine the impact of TSP and HTP on equity in health financing. Relatively significant cash became available for households after the introduction of TSP, and as a result, the FFC index was improved and the CHE index was reduced. However, over time, the harmful effects of the distribution of money and the growth of liquidity became apparent, and inflation and poverty increased sharply. The TSP increased the inflation rate and as a result, restricted the household's choices and decreased their purchasing power.

The accurate analysis of the justice index and the survey of households faced with CHE are not possible without identifying target groups and households. Therefore, it is necessary to identify households with a higher probability of Catastrophic Health Expenditure than others, according to their economic and social characteristics as far as possible. The socioeconomic characteristics of CHE households are described in detail in this study.

Since people over 65 are considered vulnerable and exposed to high costs of treatment, their presence in households has a positive and significant effect on the bearing of catastrophic health expenditures. Moreover, as the number of elderly people in households is higher, households are more likely to face CHE. The study by Ma et al. [15], Pal [26], Wyszewianski [37], Hajizadeh and Nghiem [11], and Merlis et al. [16], also confirms the result. This variable was significant and positive in both models at level 99%, which means that an increase in the elderly population in the household increases the probability of suffering from catastrophic health expenditures. The odds of this variable equal 1/31 for the presence of an aged person in the family and 1/85 for the presence of a more aged person. Households with one elderly person are 1.31 and households with more than one elderly person are 1.85 times more likely to face CHE than non-elderly families. Due to the aging population in Iran, policymakers should pay particular attention to this issue.

The risk of exposure to catastrophic health expenditures in rural areas is higher than in urban areas, which is significant at 99%; rural households are more likely to face catastrophic health expenditures. Of course, this result was expected. The residents of rural areas have lower relative incomes and, of course, health facilities are less than in urban areas.

As expected, the number of employees per household reduces the probability of CHE exposure. The negative coefficient and significance level of 99% of this variable confirmed the hypothesis that in households with more employees, the probability of households being exposed to CHE decreases. The odds ratio of 0.75 means the households with only one employee and 0.72 means more employees. This result is consistent with the studies conducted by Hajizadeh and Nghie [11, 17], Pal [26].

One of the innovations of the present study (which is not observed in previous studies) was to consider the development index of the province of the place of residence of households in terms of access to healthcare providers as a factor affecting the probability of facing CHE. According to the results, households living in Iran's less developed provinces have been more exposed to CHE health. Border provinces and provinces that are more in arid and desert climates have less prosperity and income. They also have less access to sanitation and safe drinking water. Therefore, on the one hand, they need more medical services and on the other hand, they have a low income, so they are more likely to face CHE in these provinces.

The results indicate that households living in mortgage or rental houses are more likely to suffer from CHE than those who own a home. The coefficient of this variable at the confidence level of 99% was significant and negative. The odds ratio is equal to 0.89, and because this ratio is less than one, it is interpreted that property ownership can be a household protecting variable against CHE. Ekman [4] showed that housing ownership is one of the barrier variables to household CHE healthcare exposure.

With an increase in the number of educated people in a family, the probability of household exposure to CHE decreases. Given the fact that literacy opportunities are higher in well-off families, and being literate provides more economic opportunities for individuals, literate people are also better off with lifestyles and avoid high-risk behaviors.

Although some studies such as Su et al. [31] show that the probability of CHE is higher in large households (in their study, CHE increased by 5% per person added to the household population), the results of our study differ. According to the results, CHE expenditures were relatively higher in sparsely populated households. It can be argued that in recent years, due to the deteriorating economic situation of Iranian households, most of the less well-off households, especially those without housing, have often turned to having only one child.

Insurance coverage has not reduced the probability of household exposure to CHE. This variable was significant at 99% level and its odds ratio was 1.14. Considering the mechanism of medical insurance (accumulation of risk), health insurance should be a factor in reducing the likelihood of a household facing CHE [30]. In Thailand, the implementation of insurance policies and prepaid mechanisms is considered among the most important factors in protecting households against CHE [14]. However, limited studies such as Ekman [4], Wagstaff and Lindlow [33], Ghiasvand et al. [8], Nekoeimoghadam et al. [23], and O'Donnell et al. [25] show that health insurance increases the risk of exposure to CHE for households by encouraging people to use more services as well as more advanced services:

A) Inefficiency of health insurance in terms of non-coverage of healthcare services in the sense of not defining suitable packages of services by insurance [4]
B) Increased induction demand of households and consequently the increased share of health expenditure in the household budget [33]
C) The inadequacy of the insurance coverage depth, that is, insurances cover a small share of service expenditure, and the burden of more health expenditure is placed on the shoulder of the household, which increases their risk of facing catastrophic expenditure. The studies by Rezapur et al. [28] and Faradonb et al [6] conducted in Iran, confirms the results of the present study.

Income deciles are a measure of the household's economic situation; the negative and significant effect, at a 99% confidence level, on the probability of suffering from CHE, indicates that lower deciles are more likely to suffer from CHE than households in upper deciles. The results of Su et al. [31] and Ekman [4] also confirm the results of this study. This result is important in two respects: first, due to the lack of insurance efficiency and the high share of out-of-pocket payment, lower deciles are more exposed to CHE and second, the prevalence of illness is higher in lower deciles. Usually, there are poor living facilities, low literacy levels, poor nutrition, and poor living environment in the lower-income deciles. For these reasons, all kinds of diseases and health problems are more likely to occur, and some of them do not have insurance coverage.

Based on the results, granting cash subsidies at a significant level of 99% has increased the probability of facing CHE. Moreover, because the odds ratio of this variable is more than one, it is construed that subsidies to households cannot be a protective variable for the household against household exposure to catastrophic health expenditures. A clear picture of the effect of such a plan on the CHE of Iranian households is shown, such that these expenditures have fallen sharply since 2011, and continued in 2012. However, paying cash subsidies directly to bank accounts created a significant leap in the liquidity amount of the people. Based on economic courses, the inflation growth rate is one of the most reliable outcomes of liquidity growth. Although inflation in Iran was 10.13% in 2010, it reached 20.62% in 2011 and reached 27.35% and 39.26% in 2012 and 2013, respectively. The inflation rate reached over 40% in 2019 and 2020 (based on World Bank data). Of course, inflation has been much worse for the health sector, and health sector inflation exceeded inflation in the entire economy. This situation had a quite devastating effect on the health sector in Iran. The CHE of the households exposed to these costs sharply raised since 2012, and even exceeded the pre-implementation of targeted subsidies. It can be judged that the implementation of this policy has harmed one of the most important sectors of household welfare, i.e. health.

The HTP project, with the primary goal of promoting justice in the health sector, had a heavy financial burden on the government but failed to be effective. The plan was accompanied by an intensification of the government budget deficit and high inflation and an increase in the poverty line. Thus, the household situation weakened the lower decile. The results of this study indicate that after the implementation of this plan, there has been no change in the situation of Iranian households regarding the indicators of justice in health financing unless it has prevented the deterioration of household health payments.

## Conclusion

To prevent the escalation of inequality and the situation of the poor, the long-term inflationary effects of policies must be seriously considered by politicians. During the implementation of TSP, relatively significant cash became available for households, thus improving the CHE and FFC indices over the short term. Nevertheless, afterward, the harmful effects of the distribution of money and the growth of liquidity became apparent (even households began to receive several loans from banks with the support of this money), and inflation and the poverty line increased sharply. The policy pursued by the government to reduce poverty resulted in rising inflation above 40% and the poverty line has increased from 10,800,000 Rials ($ 257) to 26,750,000 Rials ($ 636) in 2013. It also caused a sharp decline in the national currency value. The out-of-pocket payment was over 50% between 2011 and 2013 [34]. Therefore, although this plan was implemented to improve justice, there were no satisfactory results in the area of equity financing of the health sector, and we witnessed high inflation years after the implementation of this plan, caused by the injection of liquidity into the community. Therefore, there would be probably far better results if monthly cash payments for households were done as expanding insurance coverage.

Another important policy was the implementation of the costly plan for the health system transformation. Although the government claims that this plan has been successful, in the years after its implementation, we did not observe a significant change in the status of equity indices in health financing. One of the dimensions of the inefficiency of government support policies is to ignore the social and economic characteristics of households in implementing plans to reduce their chances of facing CHE.

The present study investigated the effect of other factors on the probability of exposure to CHE in households, which can provide more reliable results than previous studies given the large sample size. The presence of elderly people (over 65 years of age) increases the risk of CHE in the household. Therefore, elderly empowerment policies, as well as the modification of their insurance coverage, can protect households with the elderly against CHE. One of the important issues in Iran's economy is the high cost of housing. The share of housing in the household budget in Iran is much higher than in other countries. The reason for this is not the subject of the present study, but households that are not homeowners, due to the high rental cost of housing in Iran, spend a large part of their budget on housing costs. Therefore, it is very likely that such households will be faced with CHE. It seems necessary to reform housing laws and policies to support homeless families in Iran.

Finally, it can be stated that one of the most important means of protecting households against CHE is insurance coverage. Of course, insurance coverage in Iran has not been able to have such an effect, which is rooted in the inadequacy of insurance. Typically, insurance in Iran is defined for effective and poor classes. In Iran, wages are not aligned with the price and inflation, which is not a distant thought. This result is one of the most significant results of this article, which demonstrates the ineffectiveness of basic insurance. Consequently, basic insurance is inefficient in Iran. Modifying the structure of insurance and improving its efficiency should be a top priority of the health sector. In addition, according to the results of this study, households without housing, low income, elderly members, and rural and living in less developed provinces should be among the priorities of government protection policies. Of course, the government has recently sought to eliminate the subsidies of more well-off people and support more families at risk. The results of this article can be useful for selecting target groups.

Future economics and healthcare reforms in Iran should not only focus on expanding the coverage but also on improving the equity of distribution of healthcare benefits. Government should consider the equitable distribution of subsidies, mainly among low-income citizens.

## Data Availability

The datasets generated and/or analyzed during the current study are available here: https://www.amar.org.ir/.

## Abbreviations

TSP: Targeted subsidy plan
HTP: Health Transformation Plan
FFC: Fairness in Financial Contribution
CHE: Catastrophic Health Expenditure*s*
WHO: World Health Organization
NGO: Non-government organization
MOHME: Ministry of Health and Medical Education
PHC: Primary healthcare
OOP: Out-of-pocket
ISC: Iran Statistical Center
SSO: Social Security Organization
AFMSO: Armed Forces Medical Service Organization
IHIO: Iran Health Insurance Organization
FFC: Fair financial contribution
VAT: Value-added tax

## Footnotes

1 The statistical center's questionnaires have a different sample of households every year, so we don't deal with the same family every year. This information is not a time series and is actually a pooling, not a time series. The data has been separated into two separate parts before and after the purposeful plan, with a separate collection for each part. The reason for this type of analysis is the use of vast amounts of data for analysis.

2 The provinces are divided into three sections: deprived provinces, semi-developed provinces, and developed provinces (For more information refer to Pourasghar Sangachin et al. [27]).

3 Tithe refers to income measured by household expenses, not household income; because the potential for error increases when reporting household income, but the likelihood of error when reporting expenditures is very low, our usage involves the household expenditure decile that is also documented in the Iranian Statistics Center. The expenditure tithe is more realistic than the income tithe, as it is superior to the income tithe and more realistic.

4 Elders over 65 years old were considered.
